# Development of excitation power-responsive anti-stokes emission wavelength switching and their energy saving induced by localized surface plasmon resonance

**DOI:** 10.1186/s11671-024-03991-0

**Published:** 2024-03-14

**Authors:** Jotaro Honda, Kosuke Sugawa, Koki Honma, Seiya Fukumura, Ryuzi Katoh, Hironobu Tahara, Joe Otsuki

**Affiliations:** 1 Department of Materials and Applied Chemistry, College of Science and Technology, Nihon University, Chiyoda, Tokyo 101-8308 Japan; 2 Department of Chemical Biology and Applied Chemistry, College of Engineering, Nihon University, Koriyama, Fukushima 963-8642 Japan; 3 Graduate School of Engineering, Nagasaki University, 1-14 Bunkyo, Nagasaki, 852-8521 Japan

## Abstract

We designed an external stimulus-responsive anti-Stokes emission switching using dual-annihilator-based triplet–triplet annihilation upconversion systems. This system, which was constructed by incorporating a palladium porphyrin derivative as a sensitizer and 9,10-diphenylanthracene (DPA) and 9,10-bis(triisopropylsilyl)ethynylanthracene (TIPS) as annihilators into polymer thin films, produced TIPS- and DPA-based anti-Stokes emission under low and high excitation powers, respectively. The mechanism involves the following: under low excitation power, triplet energy transfer from triplet-excited PdOEP to DPA is induced, followed by relay to TIPS. This results in the generation of triplet-excited TIPS, and the subsequent triplet–triplet annihilation between them produces TIPS-based anti-Stokes emission. Conversely, under high excitation power, the high-density triplet-excited DPA, generated through triplet energy transfer from PdOEP, undergoes triplet–triplet annihilation among themselves, resulting in the generation of DPA-based anti-Stokes emission. Additionally, we achieved energy savings by reducing the required excitation power for switching through the utilization of plasmonic metal nanoparticles. The strong local electromagnetic fields associated with the localized surface plasmon resonance of metal nanoparticles enhance the photoexcitation efficiency of PdOEP, subsequently increasing the density of triplet-excited DPA. As a result, anti-Stokes emission switching becomes feasible at lower excitation powers.

**Supplementary Information:**

The online version contains supplementary material available at 10.1186/s11671-024-03991-0.

## Introduction

A class of smart emissive materials can change its emission color (wavelength) in response to a variety of external stimuli, including mechanical force [1], light [2, 3], electric field [4, 5], organic solvent (vapor) [6], heat [7, 8], humidity [9], pH [10], and so on. These materials have garnered significant interest due to their diverse applications, including chemical sensing of volatile organic compounds (VOCs)[11] and biomacromolecules [12], nanothermometry [13], advanced anti-counterfeiting technologies [14], bionanoprobes [15], biosensors [16], and optical logic gates.[17] Advancements in these smart emissive materials are expected to greatly influence the development of next-generation optical technologies. One promising direction for future development is the creation of emissive materials capable of switching in anti-Stokes emission.

The anti-Stokes emission, which is characterized by shorter wavelengths than the excitation wavelength, is a unique phenomenon found in photon upconversion systems that convert long-wavelength (low-energy) light into short-wavelength (high-energy) light. This property holds significant promise for various applications. For example, long-wavelength excitation light can deeply penetrate biological tissues, making it valuable for biological applications [18]. Additionally, the unusual anti-Stokes emission does not interfere with the background autofluorescence (Stokes emission) from biological substrates, rendering it ideal for the development of intelligent emissive materials used in bionanoprobe technology [19].

Among various photon upconversion techniques [20, 21], triplet–triplet annihilation (TTA)-based upconversion (TTA-UC) systems stand out for their versatility. TTA-UC systems, consisting of a sensitizer and an annihilator, offer tunable excitation and emission wavelengths through specific sensitizer-annihilator combinations [22]. Recently, there has been a surge in materials and devices capable of switching anti-Stokes emission, primarily within TTA-UC systems. These switching functions can be categorized into two types. Type I involves ON/OFF switching of anti-Stokes emission, which is achieved through external stimuli including light [23–26], heat [27], and others [28], sometimes affecting Stokes emission (sensitizer phosphorescence) as well [29–32]. Type II, conversely, focuses on changing the wavelength of anti-Stokes emission. While some success has been reported with heat-induced liquid–crystal/crystal phase transitions in annihilators[33] and chemical stimulus-induced protonation/deprotonation of annihilators [34], it remains an area with limited accomplishments. Therefore, the development of Type II smart emissive materials that respond to external stimuli by skillfully controlling the TTA-UC optical transitions becomes crucial.

In the meantime, it has been found that plasmonic metal nanoparticles can significantly impact the optical behavior of nearby photofunctional molecules and optical systems. This effect is not an exception in the context of TTA-UC systems [35, 36]. The localized surface plasmon (LSP) resonance of the metal nanoparticles generates strong local electromagnetic fields, which have several consequences [37]. These fields enhance the photoexcitation rate of phosphorescent sensitizers [38, 39]. Additionally, the strong local electromagnetic fields accelerate the radiative decay rates from the singlet- and triplet-excited sensitizers, [38, 40] as well as singlet-excited emitters [38], known as the Purcell effect. Furthermore, LSP resonance and/or metal nanoparticles effectively quench the triplet-excited annihilator [41]. By strategically utilizing these optical effects, the anti-Stokes shift switching could be driven by lower-power input light.

In this study, we have developed smart emissive systems, which enable the switching of anti-Stokes emission wavelengths by manipulating the excitation power. These systems are based on unique dual-annihilator-based TTA-UC systems. The distinct switching mechanism relies on the excitation power-dependent branching of the deactivation pathway of the triplet-excited energy, which is transferred from the sensitizer to one of the annihilators. Moreover, the incorporation of anisotropic plasmonic metal nanoparticles into the systems has been demonstrated to lower the necessary excitation power for the switching process, resulting in energy savings.

## Results and discussion

**Fig. 1:**
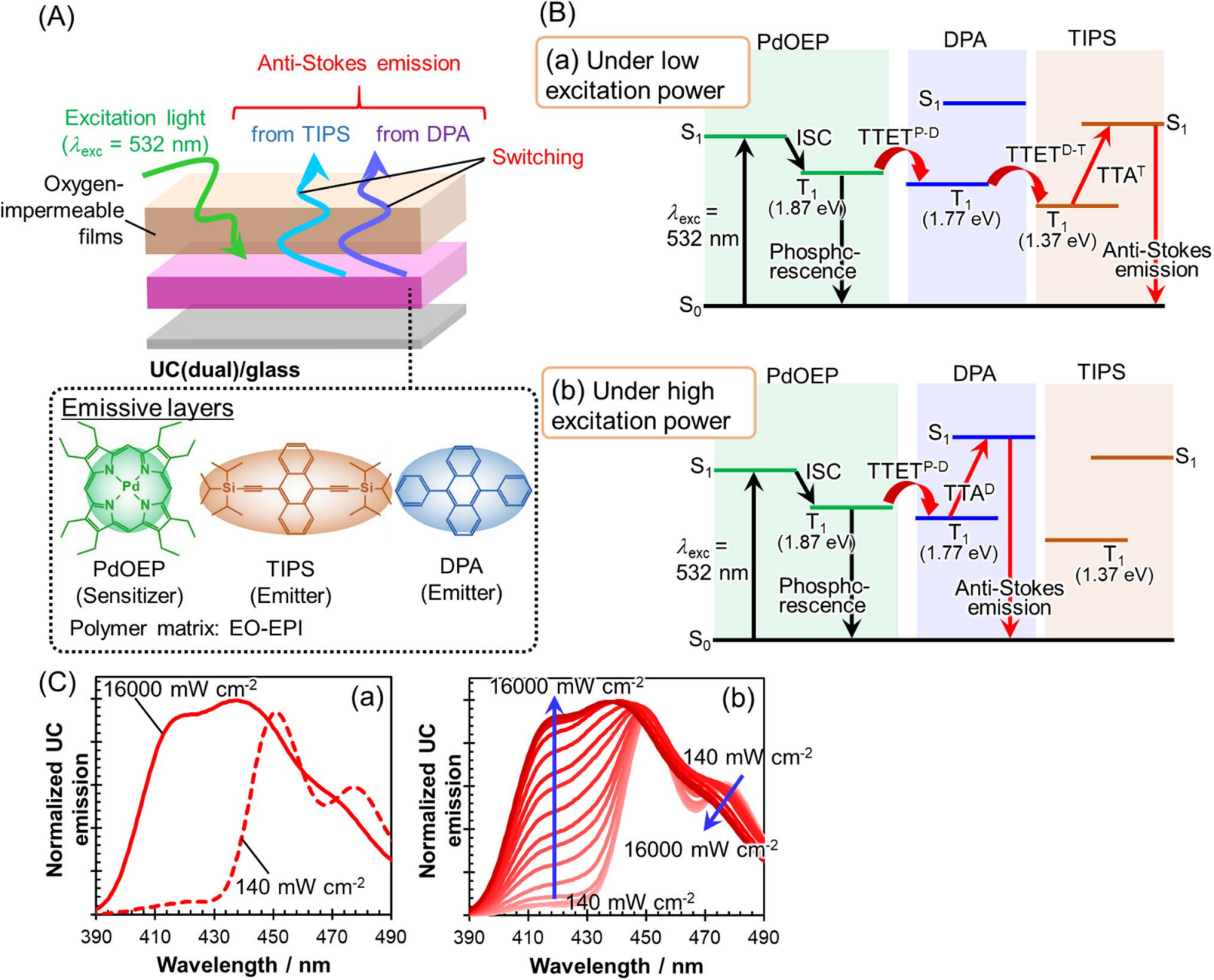
**A** Schematic showing of anti-Stokes emission switching systems (UC(dual)/glass). **B** Proposed emission mechanisms of (a) TIPS-based anti-Stokes emission under low excitation power and (b) DPA-based anti-Stokes emission under high excitation power. **C** (a) Anti-Stokes emission spectra under continuous 532 nm laser excitation at 140 and 16,000 mW cm^−2^. (b) Excitation power dependence (140–16000 mW cm^−2^) of the anti-Stokes emission spectrum

The schematic drawing of our developed smart emissive systems is shown in Fig. 1A. The emissive solid thin film (average thickness: 15 nm, Additional file 1: Fig. S1) is made of ethylene oxide-epichlorohydrin copolymer (EO-EPI) containing palladium(II) octaethylporphyrin (PdOEP) as a sensitizer and dual annihilators, 9,10-diphenylanthracene (DPA) and 9,10-bis(triisopropylsilyl)ethynylanthracene (TIPS). It was confirmed from the absorption spectrum (Additional file 1: Fig. S2) that the three elements for generating anti-Stokes emission were contained in the thin films. These films were coated with an oxygen-impermeable polyvinyl alcohol derivative (Exceval™, Kuraray) to hinder the quenching of triplet excitons by oxygen in the anti-Stokes emission process. This system is referred to as UC(dual)/glass. The detailed fabrication procedures for UC(dual)/glass and the reference samples consisting of mono-annihilator-based TTA-UC thin films can be found in Materials and Methods.

We envisioned the following mechanistic scenario in which the anti-Stokes shift can be switched by manipulating the excitation power. When exposed to low excitation power irradiation (Fig. 1B(a)), the triplet-excited energy generated through intersystem crossing (ISC) from the singlet-excited PdOEP is transferred via TTET^P−D^ to generate the triplet-excited DPA. Since the excitation power is low, so is the density of triplet-excited DPA. Therefore, it is more probable that this energy transfers through TTET^D−T^ to abundant ground state TIPS rather than being utilized for TTA^D^ among the triplet-excited DPA. Subsequently, collisions between the triplet-excited TIPS produce the singlet-excited TIPS through TTA^T^. Consequently, it is expected that the anti-Stokes emission within the 440–500 nm range would be emitted, corresponding to the TIPS fluorescence. On the other hand, under high excitation power irradiation (Fig. 1B(b)), a significant number of triplet-excited PdOEP molecules are expected to concurrently transfer triplet-excited energy to DPA, resulting in the formation of a high-density triplet-excited DPA. As a result, there will be a higher chance of TTA (TTA^D^) between the triplet-excited DPA molecules themselves rather than TTET^D−T^,[42] ultimately resulting in the formation of singlet-excited DPA. The outcome would be the generation of anti-Stokes emission corresponding to the DPA fluorescence within a 390–470 nm range.

To experimentally validate our proposed anti-Stokes emission wavelength switching, we investigated the excitation power dependence (532 nm laser for excitation of the Q-band of PdOEP, 140–16000 mW cm^−2^) of the anti-Stokes emission spectra for UC(dual)/glass as well as for the mono-annihilator-based TTA-UC thin films, UC(DPA)/glass and UC(TIPS)/glass. For mono-annihilator films (the absorption spectra are shown in Additional file 1: Fig. S2), the anti-Stokes emission from the respective annihilator contained in the film was observed irrespective of the excitation power, i.e., 390–470 nm from UC(DPA)/glass and 440–500 nm from UC(TIPS)/glass as shown in Additional file 1: Fig. S3. The origin of the emission was identified unequivocally by comparing it with the fluorescence spectra of these compounds in solutions, as shown in Additional file 1: Fig. S4. The process involves the TTET from the triplet-excited PdOEP to the annihilator, followed by TTA between the triplet-excited annihilators, leading to the formation of singlet-excited annihilators [43]. The anti-Stokes emission efficiencies of UC(DPA)/glass and UC(TIPS)/glass were estimated to be 0.003 and 0.005, respectively (Additional file 1: Table S1). The singlet-excited and triplet-excited lifetimes of each annihilator are also described in the Additional file 1: Fig. S5. In sharp contrast, UC(dual)/glass displayed clear switching behavior between the anti-Stokes emissions from DPA and TIPS dependent on the excitation power, as shown in Fig. 1C. When the irradiation power is low (140 mW cm^−2^), the anti-Stokes emission predominantly originated from the TIPS fluorescence, as judged from the spectral shape. As the excitation power increased, the contribution of DPA fluorescence progressively increased. With the largest excitation power (16,000 mW cm^−2^), the anti-Stokes emission was dominated by the DPA fluorescence. These results clearly demonstrate that we successfully implemented the anti-Stokes emission switching, which can be controlled by modulating the excitation power.

**Fig. 2:**
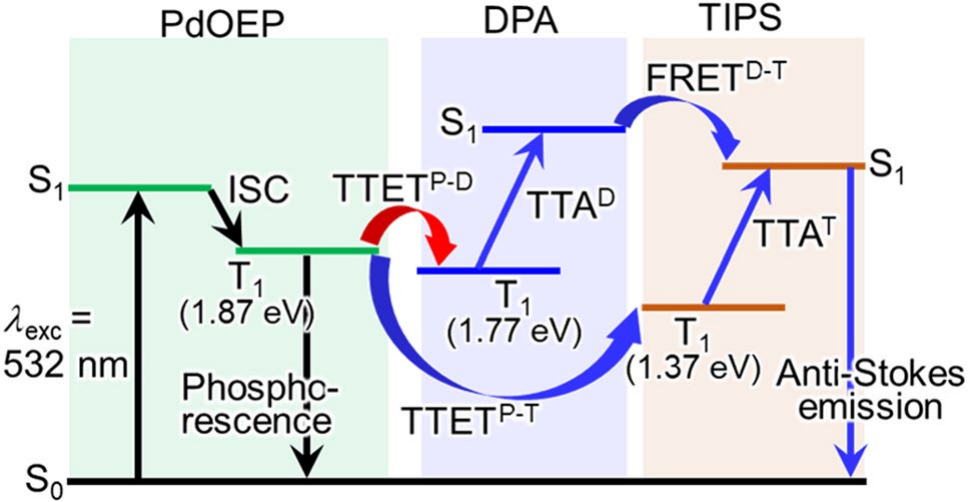
Optical transitions that could be induced besides the mechanisms described in Fig. 1

Under high-power excitation conditions, in which anti-Stokes emission from DPA is predominant, the mechanism we envisioned and illustrated in Fig. 1B(b) is reasonable in line with the established TTET-TTA systems. The mechanism under low-power excitation, however, requires a more scrutiny because, in principle, there could be other pathways resulting in the generation of the singlet-excited TIPS than we initially envisioned (Fig. 1B(a)). These possible pathways include the direct triplet–triplet energy transfer from triplet-excited PdOEP to TIPS (TTET^P−T^) and the Förster-type singlet–singlet energy transfer from the singlet-excited DPA to TIPS. as depicted in Fig. 2.

To address the possibility of TTET^P−T^, the efficiencies of TTET^P−D^ and TTET^P−T^ were estimated using the mono-annihilator model systems, UC(DPA)/glass and UC(TIPS)/glass. To efficiencies of TTET^P−D^ (${\Phi }_{{\text{TTET}}}^{{\text{P}}-{\text{D}}}$) for UC(DPA)/glass and TTET^P−T^ (${\Phi }_{{\text{TTET}}}^{{\text{P}}-{\text{T}}}$) for UC(TIPS)/glass were estimated based on Eq. (1) (Additional file 1).

1 $${\Phi }_{{\text{TTET}}}^{{\text{P}}-{\text{D}}(\text{or P}-{\text{T}})}=1-\frac{{I}_{{\text{phos}}}^{{\text{UC}}({\text{DPA}})\left(\text{or} \text{UC}({\text{TIPS}})\right)}}{{I}_{{\text{phos}}}^{{\text{PdOEP}}}},$$

in which ${I}_{{\text{phos}}}^{{\text{UC}}({\text{DPA}})}$, ${I}_{{\text{phos}}}^{{\text{UC}}({\text{TIPS}})}$, and ${I}_{{\text{phos}}}^{{\text{PdOEP}}}$ are the phosphorescence intensities of UC(DPA)/glass, UC(TIPS)/glass, and PdOEP thin films without any annihilators (PdOEP/glass) (Additional file 1: Fig. S6) [41]: We obtained the values of ${\Phi }_{{\text{TTET}}}^{{\text{P}}-{\text{D}}}$ = 0.996 and ${\Phi }_{{\text{TTET}}}^{{\text{P}}-{\text{T}}}$ = 0.418. Assuming that the triplet energy transfer from PdOEP to DPA and TIPS in UC(dual)/glass occurred at the same rate as in UC(DPA)/glass and UC(TIPS)/glass, respectively, the efficiency of TTET^P−T^ (${\Phi }_{{\text{TTET}}}^{{\text{P}}-{\text{T}}({\text{dual}})}$) in UC(dual)/glass was estimated to be negligibly small (0.029) compared to that of TTET^P−D^ (${\Phi }_{{\text{TTET}}}^{{\text{P}}-{\text{D}}({\text{dual}})}$= 0.968), as calculated in Additional file 1. The direct TTET from the triplet-excited PdOEP to TIPS is negligible in UC(dual)/glass. The significantly lower ${\Phi }_{{\text{TTET}}}^{{\text{P}}-{\text{T}}({\text{dual}})}$ compared to ${\Phi }_{{\text{TTET}}}^{{\text{P}}-{\text{D}}({\text{dual}})}$ is attributed to the lower concentration of TIPS in UC(dual)/glass compared to that of DPA. The concentrations of PdOEP, DPA, and TIPS were estimated to as 11.5 mM, 380 mM, and 140 mM, respectively (the detailed estimation method is described in the Additional file 1: Fig. S7). Since the DPA concentration was 2.7 times higher than that of TIPS, TTET^P−D^ is expected to be favored over TTET^P−T^. This estimation is consistent with the predominant observation of DPA fluorescence under high-power excitation conditions. The values of ${\Phi }_{{\text{TTET}}}^{{\text{P}}-{\text{T}}({\text{dual}})}$ and ${\Phi }_{{\text{TTET}}}^{{\text{P}}-{\text{D}}({\text{dual}})}$ in UC(dual)/glass should be independent of the excitation power. Therefore, if the direct pathway TTET^P−T^ significantly contributed, TIPS-based anti-Stokes emission should have also been observed not only under low excitation power but also under high excitation power conditions. A similar reasoning can apply to dismiss the other possibility of the FRET^D−T^. If the FRET^D−T^, which should be independent of the excitation power, ever contributed significantly, TIPS-based anti-Stokes emission should have been also observed under high excitation power conditions. Indeed, as shown in Additional file 1: Fig. S8, the efficiency of FRET^D−T^ was confirmed to be quite low, approximately 20%, from the fluorescence spectra of UC(DPA)/glass and UC(dual)/glass. This low efficiency of FRET^D−T^ may be attributed to the low concentration of TIPS, the acceptor of singlet-excited energy from DPA. These considerations establish the processes shown in Figs. 1B(a) and (b) as the mechanism of our anti-Stokes switching by means of the excitation power modulation.

**Fig. 3:**
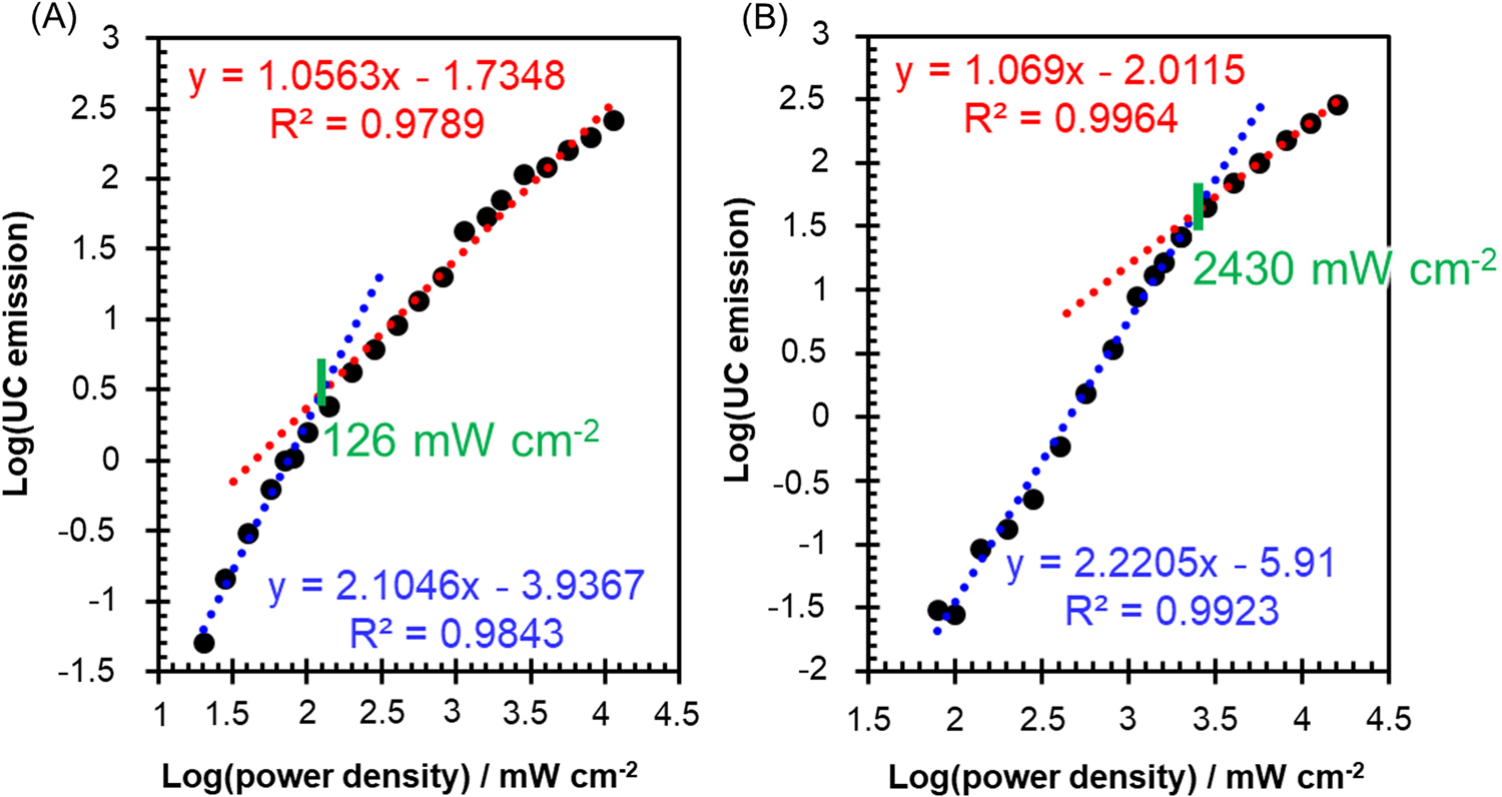
Double-logarithmic plots of anti-Stokes emission intensity at 418 nm, corresponding to DPA-based anti-Stokes emission, as a function of 532 nm laser excitation power for **A** UC(DPA)/glass and **B** UC(dual)/glass to estimate *I*_th_

Next, we estimated the power density threshold (*I*_th_) for UC(DPA)/glass and UC(dual)/glass. While the anti-Stokes emission intensity from TTA-UC systems increases quadratically under low excitation power irradiation, it increases linearly under high excitation power irradiation. These correlations give rise to two linear regions with gradients of two and one, respectively, on the double-logarithmic plot of the anti-Stokes emission intensities against the excitation power. The excitation power at the crossing point of these regions is defined as the threshold power *I*_th_.[43, 44] At this excitation power, the rate of triplet deactivation of the annihilator through TTA matches the rate of spontaneous triplet deactivation. In this study, we determined the *I*_th_ for the anti-Stokes emission attributed to DPA by measuring the emission intensity at 418 nm, in which TIPS-based anti-Stokes emission was minimal. As shown in Fig. 3, UC(dual)/glass exhibited an *I*_th_ of 2430 mW cm^−2^, which was significantly higher than that (126 mW cm^−2^) of UC(DPA)/glass. The value of ${I}_{{\text{th}}}(={\left({\gamma }_{{\text{exc}}}{\Phi }_{{\text{TTET}}}{\gamma }_{{\text{TT}}}{\left({\tau }_{{\text{T}}}^{0}\right)}^{2}\right)}^{-1})$[44] depends on the excitation efficiency (${\gamma }_{{\text{exc}}}$), TTET efficiency from the triplet-excited sensitizer to annihilator (${\Phi }_{{\text{TTET}}}$), second-order annihilation constant (${\gamma }_{{\text{TT}}}$), and triplet state lifetime of the annihilator (${\tau }_{{\text{T}}}^{0}$). Since both samples employ the same sensitizer (PdOEP) and annihilator (DPA), it is reasonable to assume that ${\gamma }_{{\text{exc}}}$ and ${\gamma }_{{\text{TT}}}$ are the same for both samples. Moreover, the TTET efficiencies in these samples are nearly the same, as calculated in Additional file 1, *i.e.*, ${\Phi }_{{\text{TTET}}}^{{\text{P}}-{\text{D}}}$ = 0.996 and ${\Phi }_{{\text{TTET}}}^{{\text{P}}-{\text{D}}({\text{dual}})}$ = 0.968. Consequently, the noticeable variation in *I*_th_ is likely attributed to differences in ${\tau }_{{\text{T}}}^{0}$ of DPA between the two samples. Specifically, ${\tau }_{{\text{T}}}^{0}$ of UC(DPA)/glass (${\tau }_{{\text{T}}}^{0,{\text{D}}}$) is determined by ${\tau }_{{\text{T}}}^{0,{\text{D}}}=1/{k}_{{\text{nr}}}^{{\text{D}}}$, where ${k}_{{\text{nr}}}^{{\text{D}}}$ represents the nonradiative decay rate of triplet-excited DPA. In contrast, for UC(dual)/glass, the ${\tau }_{{\text{T}}}^{0}$ (${\tau }_{{\text{T}}}^{0,{\text{D}}({\text{dual}})}$) should follow ${\tau }_{{\text{T}}}^{0,{\text{D}}({\text{dual}})}=1/({k}_{{\text{nr}}}^{{\text{D}}}+{k}_{{\text{TTET}}}^{{\text{D}}-{\text{T}}})$, as proposed in Fig. 1B(a). This suggests that the efficient TTET^D−T^ leads to a shorter ${\tau }_{{\text{T}}}^{0,{\text{D}}({\text{dual}})}$ compared to ${\tau }_{{\text{T}}}^{0,{\text{D}}}$. Therefore, the significantly higher *I*_th_ of UC(dual)/glass compared to UC(DPA)/glass aligns with our proposed mechanism, which involves the efficient TTET^D−T^. Furthermore, the anti-Stokes emission intensity exhibited an almost unitary slope with the excitation power beyond *I*_th_ (above 2800 mW cm^−2^) in the double-logarithmic plot of UC(dual)/glass. This signifies the saturation of the TTA^D^ efficiency (${\Phi }_{{\text{TTA}}}^{{\text{D}}}$) between triplet-excited DPA. Consequently, it is suggested that the primary deactivation pathway from triplet-excited DPA is dominated by TTA^D^ rather than TTET^D−T^ in this power range, which provides substantial support for the proposed mechanism as described in Fig. 1B(b).

**Fig. 4:**
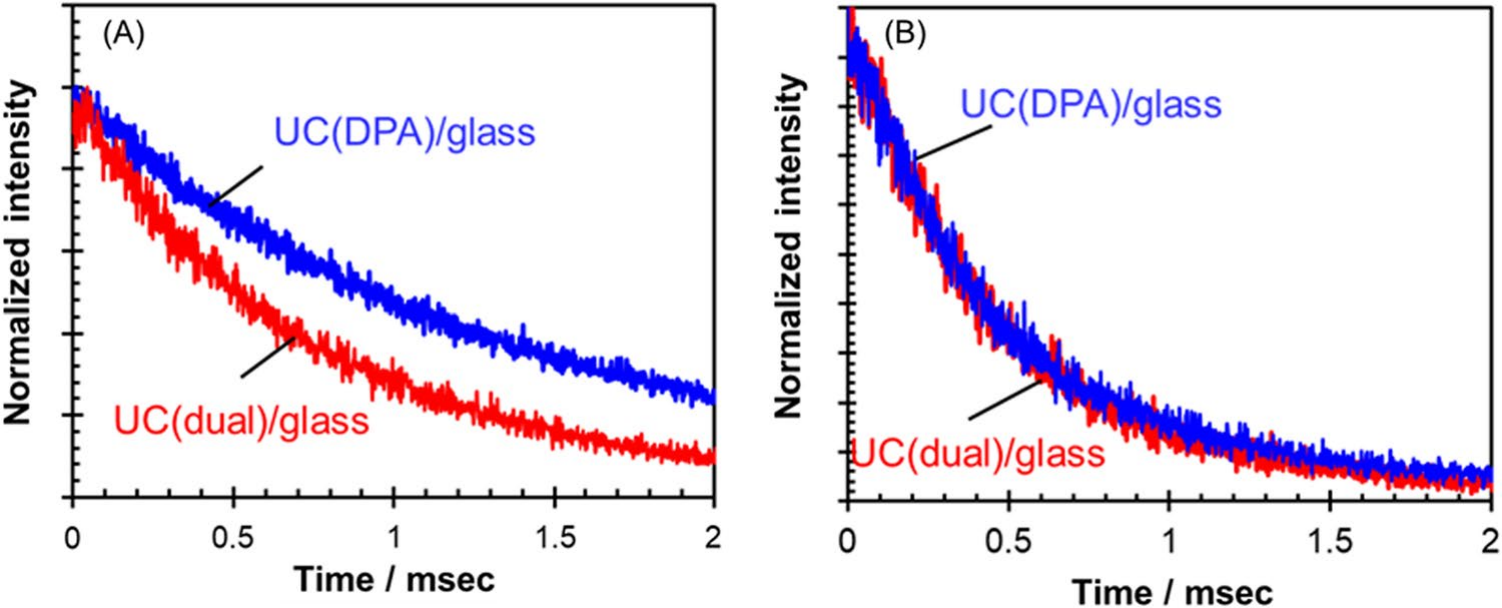
Time-resolved DPA-based anti-Stokes emission (*λ*_exc_ = 532 nm) in UC(DPA)/glass and UC(dual)/glass with excitation powers of **A** 100 mW cm^−2^ and **B** 100,000 mW cm^−2^. Anti-stokes emission of DPA was detected at 420 nm

To further substantiate our proposed mechanisms, we conducted time-resolved anti-Stokes emission measurements (*λ*_exc_ = 532 nm) for UC(dual)/glass and UC(DPA)/glass. The DPA fluorescence at 420 nm emitted with a low excitation power of 100 mW cm^−2^ was analyzed (Fig. 4A). The anti-Stokes emission lifetime for UC(DPA)/glass was determined to be 1.5 ms. With the estimated values of ${\Phi }_{{\text{TTET}}}^{{\text{P}}-{\text{D}}}$ (0.996) and the lifetime of triplet-excited PdOEP (490 μs, Additional file 1: Fig. S9), the phosphorescence lifetime of PdOEP in UC(DPA)/glass was calculated to be 2 μs (Additional file 1) [45]. Moreover, DPA exhibit a fluorescence lifetime generally less than 10 ns [46]. Thus, the extended emission lifetime is attributed to the slow (gradual) conversion from the triplet-excited to singlet-excited annihilators, which aligns with the intrinsic long lifetime of triplet-excited DPA (1–5 ms) [47]. On the flip side, the anti-Stokes emission lifetime of DPA in UC(dual)/glass was notably shorter (0.9 ms) than that in UC(DPA)/glass. The triplet lifetime (${\tau }_{{\text{T}}}^{{\text{D}}}$) of DPA in UC(DPA)/glass follows the equation ${\tau }_{{\text{T}}}^{{\text{D}}}=1/({k}_{{\text{nr}}}^{{\text{D}}}+{k}_{{\text{TTA}}}^{{\text{D}}})$ (note that the definition of ${\tau }_{{\text{T}}}^{{\text{D}}}$ differs from that of ${\tau }_{{\text{T}}}^{0,{\text{D}}}$ mentioned earlier). The triplet lifetime (${\tau }_{{\text{T}}}^{{\text{D}}({\text{dual}})}$) of DPA in UC(dual)/glass, on the other hand, should follow the equation ${\tau }_{{\text{T}}}^{{\text{D}}({\text{dual}})}=1/({k}_{{\text{nr}}}^{{\text{D}}}+{k}_{{\text{TTET}}}^{{\text{D}}-{\text{T}}}+{k}_{{\text{TTA}}}^{{\text{D}}})$, if our proposed mechanism shown in Fig. 1B(a) is in operation. The significantly reduced DPA triplet lifetime in UC(dual)/glass under a low excitation power indicates that TTET^D−T^ occurs quite efficiently.

The triplet-excited lifetime of TIPS in UC(dual)/glass (detected wavelength: 480 nm), estimated from the anti-Stokes emission lifetime (Additional file 1: Fig. S10), was compared with that of UC(TIPS)/glass (Additional file 1: Fig. S5). The anti-Stokes emission lifetime of UC(dual)/glass (1.0 ms) was longer than that of UC(TIPS)/glass (0.6 ms). Furthermore, using Equation (S2), the triplet-excited lifetime of TIPS in UC(dual)/glass (${\tau }_{{\text{T}}}^{0,{\text{T}}({\text{dual}})}$, 2.7 ms) was confirmed to be longer than that of UC(TIPS)/glass (${\tau }_{{\text{T}}}^{0,{\text{T}}}$, 1.1 ms). These lifetimes are apparent values in the upconversion films that might be affected by the rate of formation of the triplet state. The longer ${\tau }_{{\text{T}}}^{0,{\text{T}}({\text{dual}})}$ implies that UC(dual)/glass undergoes a more time-consuming intricate process than the simple energy transfer from PdOEP to TIPS that occurs in UC(TIPS)/glass. This aligns with the mechanism we proposed (Fig. 1B(a), energy transfer from PdOEP to TIPS via energy transfer from PdOEP to DPA.

We measured the anti-Stokes emission lifetime of DPA in UC(dual)/glass and UC(DPA)/glass with a high excitation power (100,000 mW cm^−2^) (Fig. 4B). The lifetime of UC(DPA)/glass (0.64 ms) was significantly shorter than with a low excitation power of 100 mW cm^−2^ (1.5 ms). Considering that ${k}_{{\text{nr}}}^{{\text{D}}}$ is independent of the excitation power in principle, this shortened lifetime suggests an increase in TTA^D^ rate due to the enhanced density of triplet-excited DPA. Furthermore, it is noteworthy that, while there was a significant difference in the anti-Stokes emission lifetime between UC(dual)/glass and UC(DPA)/glass at 100 mW cm^−2^, there was not a substantial difference between the two samples when excited with a high power density at 100,000 mW cm^−2^. These results suggest that the TTA^D^ rate so dominates in the decay pathways under the strong excitation that the presence/absence of TTET^D−T^ contributes only minimal influence on the lifetime, which is consistent with the proposed mechanism at high excitation power conditions (Fig. 1B(b)).

**Fig. 5:**
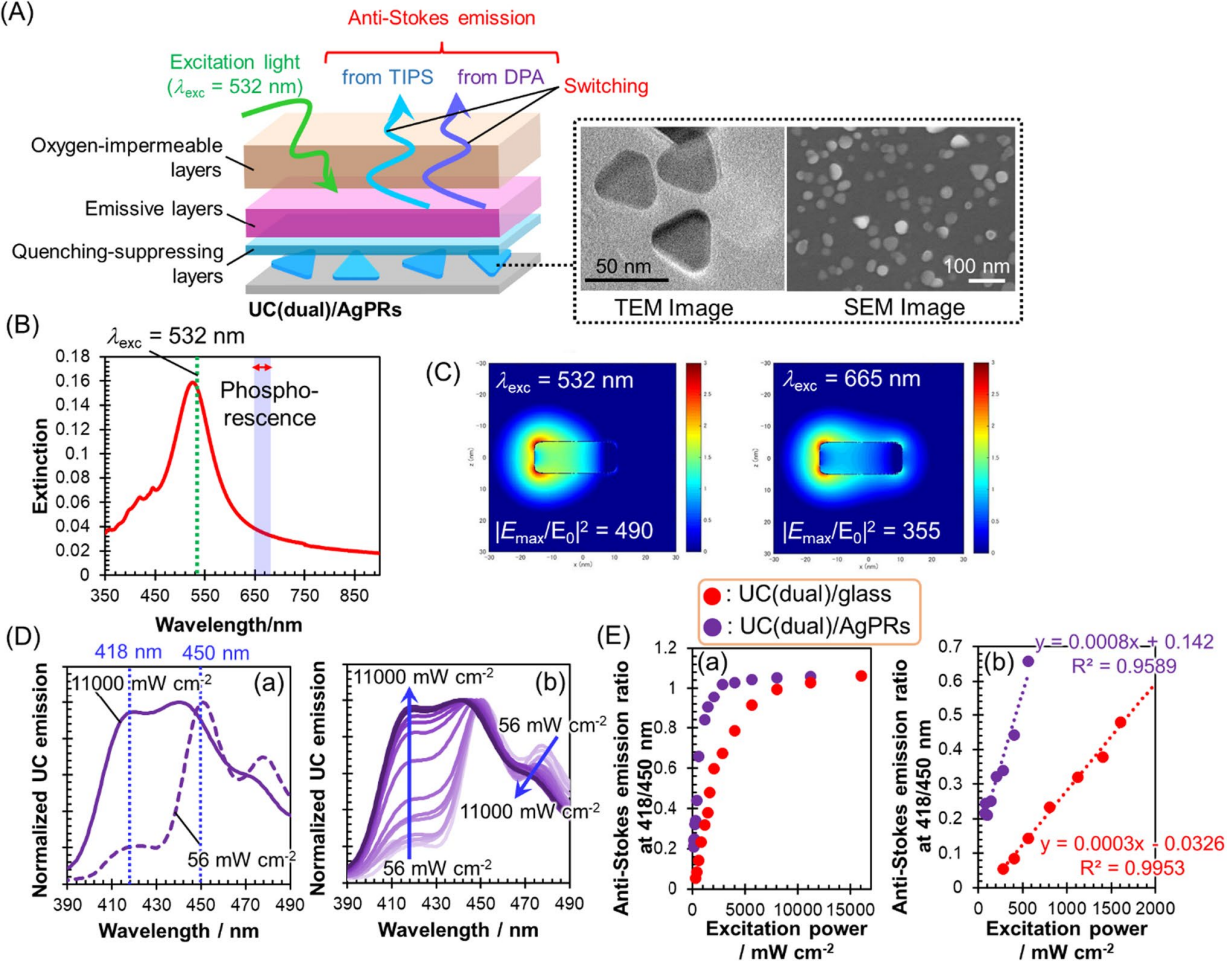
**A** Schematic showing of plasmonic anti-Stokes emission switching systems (UC(dual)/AgPRs). **B** Extinction spectrum of UC(dual)/AgPRs. **C** Electromagnetic field contour of AgPRs at 532 nm and 665 nm calculated using BEM. **D** Anti-Stokes emission spectra under continuous 532 nm laser excitation at 56 and 11,000 mW cm^−2^. (b) Excitation power dependence (56–11,000 mW cm^−2^) of the anti-Stokes emission spectrum. **E** (a) Plots of anti-Stokes emission ratio at 418 nm/450 nm against excitation power for UC(dual)/glass and UC(dual)/AgPRs. (b) Details of the linear approximation of the excitation power range

Finally, we attempted to reduce the excitation power required for the anti-Stokes emission switching by utilizing LSP resonance to decrease the energy required for the switching. The strong local electromagnetic fields generated around the plasmonic metal nanoparticles through the excitation of their LSP resonance can enhance the photoexcitation efficiency of the sensitizer, *i.e.*, PdOEP, situated in the enhanced field regions. This, in turn, would increase the population of triplet-excited DPA through ISC and TTET^P−D^ processes. Consequently, it is anticipated that the crossing from the low-power mechanism (Fig. 1B(a)) to the high-power mechanism (Fig. 1B(b)) occurs at a lower excitation power levels compared to UC(dual)/glass *without* metal nanoparticles. A Schematic drawing of the developed plasmonic anti-Stokes emission switching system is shown in Fig. 5A. Silver nanoprisms (AgPRs) with an aspect ratio (edge length/thickness) of 3.0, serving as plasmonic nanoparticles, were immobilized on a glass substrate. This immobilization was achieved with a 26 ± 1% coverage, utilizing electrostatic attraction between the negatively charged AgPRs and a glass substrate modified with positively charged polyethyleneimine (PEI) [48]. Figure 5A shows a transmission electron microscopy (TEM) image of AgPRs and a scanning electron microscopy (SEM) image of the glass substrate coated with AgPRs. The substrate was coated with stacked layers of PEI and poly(sodium 4-styrenesulfonate) (PSS) with a total thickness of 5.2 nm as quenching-suppressing layers [41], followed by a coating with EO-EPI thin films containing PdOEP and dual annihilators in the same way as UC(dual)/glass. Finally, this was coated with Exceval™ films. These are referred to as UC(dual)/AgPRs.

The PdOEP phosphorescence of UC(dual)/AgPRs at 665 nm exhibited an eightfold enhancement compared to that of UC(dual)/glass (Additional file 1: Fig. S11). It was previously reported that the phosphorescence of Pd porphyrin derivatives is boosted by a combined effect of enhanced singlet–singlet photoexcitation and radiative decay from the triplet-excited state [38, 40]. The primary LSP resonance band (in-plane dipole mode) of AgPRs closely matched the photoexcitation wavelength (532 nm) of PdOEP, but it did not align well with the phosphorescence wavelength at around 660 nm (Fig. 5B). The intensity of local electromagnetic fields (Fig. 5C), which was calculated using the boundary element method (BEM) with the geometric model illustrated in Additional file 1: Fig. S12, was significantly higher at the photoexcitation wavelength compared to the phosphorescence wavelength. These results indicate that the primary mechanism behind the phosphorescence enhancement in the present UC(dual)/AgPRs is attributed to the enhanced photoexcitation of PdOEP. In the anti-Stokes emission spectra of UC(dual)/AgPRs (Fig. 5D(a)), emissions were governed by TIPS-based and DPA-based anti-Stokes emissions under excitation powers of 56 mW cm^−2^ and 11,000 mW cm^−2^, respectively. With increasing excitation power, there was a gradual emergence of DPA-based anti-Stokes emission (Fig. 5D(b)). Thus, the switching behavior observed for UC(dual)/AgPRs is quite similar to that observed for UC(dual)/glass, implying that the underlying mechanism described above also works in the presence of AgPRs.

To investigate the effect of hybridization with AgPRs, we plotted the excitation power dependence of the anti-Stokes emission intensity ratio at 418 nm/450 nm for both UC(dual)/glass and UC(dual)/AgPRs. As shown in Fig. 5D(a), when anti-Stokes emission is dominated by TIPS-based emission, the emission intensity ratio is small. Conversely, as the contribution from DPA-based emission increases, the ratio should become larger. Consequently, UC(dual)/AgPRs reached saturation of the anti-Stokes emission intensity ratio at significantly lower excitation power (2800 mW cm^−2^) compared to UC(dual)/glass (16,000 mW cm^−2^, Fig. 5E(a)). In the low-power excitation region where the ratio is linear against the excitation power, the slope is much higher for UC(dual)/AgPRs (0.0008 per mW excitation power) than for UC(dual)/glass (0.0003), which indicates a more facile transition to the high-power mechanism region in the presence of AgPRs (Fig. 5E(b)). These results demonstrate the energy-efficient properties of anti-Stokes emission switching due to the enhancement of sensitizer photoexcitation by plasmonic local electromagnetic fields.

## Conclusions

In this study, we developed novel smart anti-Stokes emission systems capable of switching emission wavelengths in response to the excitation power. This switching mechanism cleverly utilizes the excitation power dependence of the emission mechanism of dual-annihilator-based TTA-UC systems. Moreover, it has been demonstrated that the excitation power for this switching can be controlled using plasmonic metal nanoparticles. Therefore, by adjusting the triplet energy differences between sensitizer/annihilator pairs and annihilator/annihilator pairs, as well as tuning the localized electromagnetic field intensity of LSP resonance, there is potential for purposefully controlling the switching function. Currently, our research lab is in the process of developing further unique anti-Stokes emission switching through these strategies.

## Materials and methods

### Materials

Milli-Q-grade water was utilized to prepare all aqueous solutions. Sodium tetrahydroborate (NaBH4, Tokyo Chemical Industry, Japan), trisodium citrate dihydrate (Kanto Chemical, Japan), silver nitrate (AgNO3, Fujifilm Wako Pure Chemical, Japan), sodium hydroxide (NaOH, Kishida Chemical, Japan), PEI (MW: ~ 10,000, Fujifilm Wako Pure Chemical, Japan), PSS(MW: ~ 70,000, Sigma–Aldrich, United States) ammonium solution (NH3, 28%, Kishida Chemical, Japan), hydrogen peroxide solution (H2O2, 30%, Kishida Chemical, Japan), 1,2-dichloroethane (Nacalai Tesque, Japan), PdOEP (Combi-Blocks, United States), DPA (Tokyo Chemical Industry, Japan), TIPS (Sigma-Aldrich, United States), 2-propanol (Kanto Chemical, Japan) were used as received. EO-EPI as a polymer matrix and ExcevalTM as a polyvinyl alcohol derivative were provided by Osaka Soda, Japan and Kuraray, Japan, respectively. All chemicals were used as purchased.

### Synthesis of AgPRs

First, a colloidal aqueous solution of silver nanospheres with an average diameter of 10 nm was prepared using our previously reported method [48]. To achieve this, an aqueous solution containing AgNO_3_ (1 mM, 100 mL) and trisodium citrate dihydrate (5 mM, 100 mL) was chilled in an ice bath for 1 h. Pre-chilled water was used to prepare an aqueous NaBH_4_ (20 mM, 1 mL) solution, which was then combined with the chilled solution of trisodium citrate dihydrate (5 mM, 100 mL). The resulting mixture was quickly introduced into the chilled aqueous solution mentioned earlier while being vigorously stirred. Following this, the solution was stirred for an additional 5 h, all while still in an ice bath.

The colloidal aqueous solution of AgPRs was prepared following a previously established method [49]. The pH of the colloidal aqueous solution of silver nanospheres was adjusted to 11.2 by introducing a 0.2 M NaOH aqueous solution. The solution was then exposed to light-emitting diodes (LEDs) with a center wavelength of 470 nm for 48 h to induce the formation of AgPRs.

### Preparation of sample substrates

The glass substrates with a size of 1.5 × 2.0 cm^2^ were subjected to 5-min sonication cycles in toluene, acetone, and Milli-Q water. The substrates were subsequently hydrophilized by treating them with H_2_O_2_/NH_3_ (1/1 v/v) at 100 °C for 1 h, followed by rinsing with Milli-Q water. The substrates were then immersed in an aqueous PEI solution (4.0 mg/mL) for 1 min, after which they were rinsed with Milli-Q water to be modified with PEI thin films. To prepare UC(dual)/glass, thin films of EO-EPI containing PdOEP, DPA, and TIPS were prepared on the substrate surface by spin-coating (3000 rpm, 30 s) a 1,2-dichloroethane solution containing 12.5 μM PdOEP, 625 μM DPA, 200 μM TIPS, and 0.25 wt.% EO-EPI. UC(DPA)/glass was prepared using the same method as UC/(dual)/glass. This involved using a 1,2-dichloroethane solution containing 12.5 μM PdOEP, 625 μM DPA, and 0.25 wt.% EO-EPI. Similarly, UC(TIPS)/glass was prepared following the same procedure as UC(dual)/glass, using a 1,2-dichloroethane solution containing 12.5 μM PdOEP, 200 μM TIPS, and 0.25 wt.% EO-EPI. PdOEP/glass was prepared following the same procedure as UC(dual)/glass, using a 1,2-dichloroethane solution containing 12.5 μM PdOEP and 0.25 wt.% EO-EPI. These substrates were dried for 3 h under reduced pressure. Subsequently, a 10 wt.% Exceval™ aqueous solution (150 μL) containing 5 wt.% 2-propanol was dropped onto the substrate, followed by drying them under reduced pressure for 24 h.

To prepare UC(dual)/AgPRs, the PEI-modified glass substrate was immersed in the colloidal AgPR aqueous solution for 30 min, allowing the AgPRs to immobilize the substrate surface through electrostatic attraction between the negatively charged AgPRs and the positively charged substrate surface. Spacer layers were prepared on the AgPRs-immobilized surface by immersing twice in aqueous solutions of PEI (1 mg/mL) and PSS (2 mg/mL) for 1 s each. The substrate was washed with Milli-Q water for 15 s after each immersion step. The spacer layers serve to suppress the quenching of molecular excited states by the AgPRs [40, 50]. The EO-EPI thin films containing PdOEP, DPA, and TIPS, and Exceval™ thin films were modified on the substrate surface in the same manner as UC(dual)/glass.

### Measurements

Extinction and absorption spectra were measured by a JASCO V-770 spectrophotometer. Photoluminescence spectra were measured by a JASCO FP-6500 and FP-8600. The UC emission spectra were measured under the excitation with 532 nm CW laser. The SEM observation was performed using JEOL JSM-7500FA. TEM observation was performed using a Hitachi HF-2000 system at an acceleration voltage of 200 kV. Luminescence decay profiles were measured with a custom-built time-resolved luminescence spectrometer based on the photon counting technique. A CW-YAG laser (532 nm) equipped with an optical chopper was used as a light source, and luminescence at around 420 nm was detected with a photomultiplier (Hamamatsu, R928) through bandpass filters. The arrival time of a luminescence photon was measured with a fast oscilloscope (LeCroy, HDO4104) and analyzed with a computer. After accumulation of the arrival time signals, a decay profile could be obtained.

### Supplementary Information

**Additional file 1.** Supplementary Figures and Table.

## Data Availability

The data generated during the current study are available from the corresponding author on reasonable request.
