# Supplementary material for: Development of excitation power-responsive anti-stokes emission wavelength switching and their energy saving induced by localized surface plasmon resonance
Source: Discov Nano. 2024 Mar 14;19(1):47. doi: 10.1186/s11671-024-03991-0 (PMC10940560; doi:10.1186/s11671-024-03991-0)
Supplement: Supplementary file 1 — Additional file 1. Supplementary Figures and Table. [file 11671_2024_3991_MOESM1_ESM.docx]

Development of Excitation Power-Responsive Anti-Stokes Emission Wavelength Switching and Their Energy Saving Induced by Localized Surface Plasmon Resonance

*Jotaro Honda,^1^ Kosuke Sugawa,^1,*^* *Koki Honma,^1^ Seiya Fukumura,^1^ Ryuzi Katoh,^2^ Hironobu Tahara,^3^ Joe Otsuki^1^*

^1^Department of Materials and Applied Chemistry, College of Science and Technology, Nihon University, Chiyoda, Tokyo 101-8308, Japan

^2^Department of Chemical Biology and Applied Chemistry, College of Engineering, Nihon University, Koriyama, Fukushima 963-8642, Japan

^3^Graduate School of Engineering, Nagasaki University 1-14 Bunkyo, Nagasaki 852-8521, Japan

**1. Atomic force microscopy (AFM) image of EO-EPI thin films containing PdOEP and dual annihilators**

**2. Absorption spectra of sample substrates**

**3. Excitation power dependences of UC emission spectrum for UC(DPA)/glass and UC(TIPS)/glass**

**4. Absorption and emission spectra of solutions of TTA-UC elements**

**5. The anti-Stokes emission efficiencies of UC(DPA)/glass and UC(TIPS)/glass.**

**6. Phosphorescence spectra and calculation of TTET efficiencies of sample substrates**

**7. Determination of concentration for TTA-UC components contained in UC(dual)/glass**

**8. Estimation of FRET^D-T^ efficiencies of UC(dual)/glass**

**9. Estimation of the phosphorescence lifetime of PdOEP in UC(DPA)/glass**

**10. Time-resolved TIPS-based anti-Stokes emission in UC(dual)/glass**

**11. Phosphorescence spectra of UC(dual)/AgPRs and UC(dual)/glass**

**12. Geometric models of AgPRs used in BEM calculations**

**1. Atomic force microscopy (AFM) image of EO-EPI thin films containing PdOEP and dual annihilators**


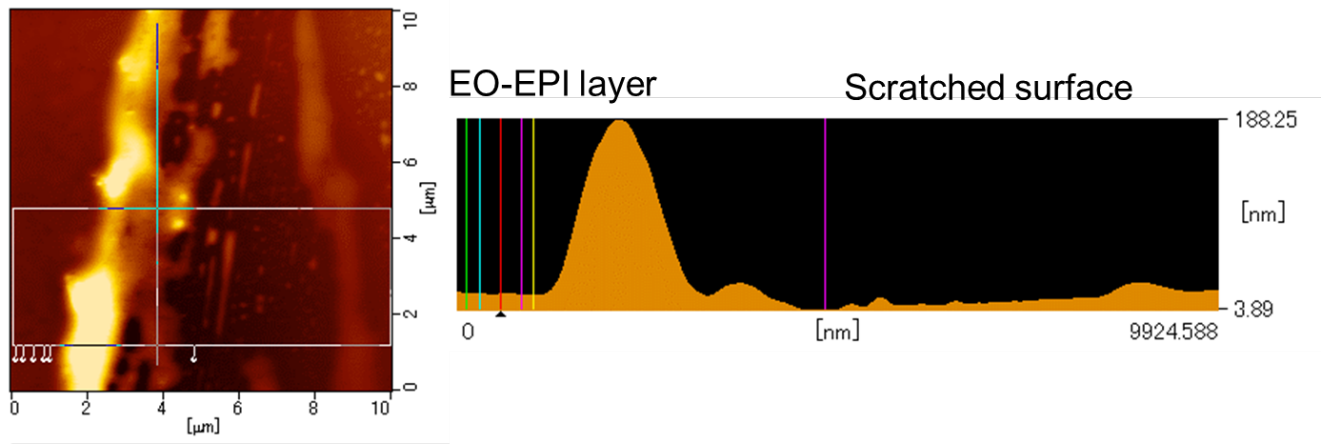


**Figure S1**. Atomic force microscopy (AFM) image of EO-EPI thin films containing PdOEP and dual annihilators.

**2. Absorption spectra of sample substrates**


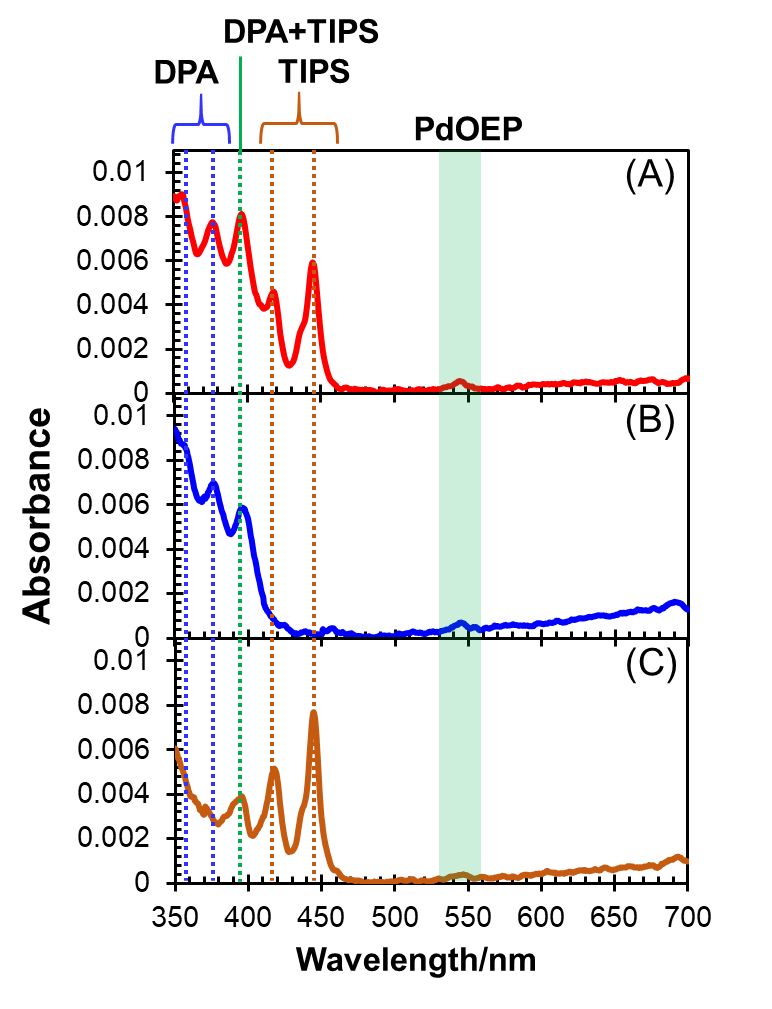


**Figure S2**. Absorption spectra of (A) UC(dual)/glass, (B) UC(DPA)/glass, and (C) UC(TIPS)/glass.

**3. Excitation power dependences of UC emission spectrum for UC(DPA)/glass and UC(TIPS)/glass**


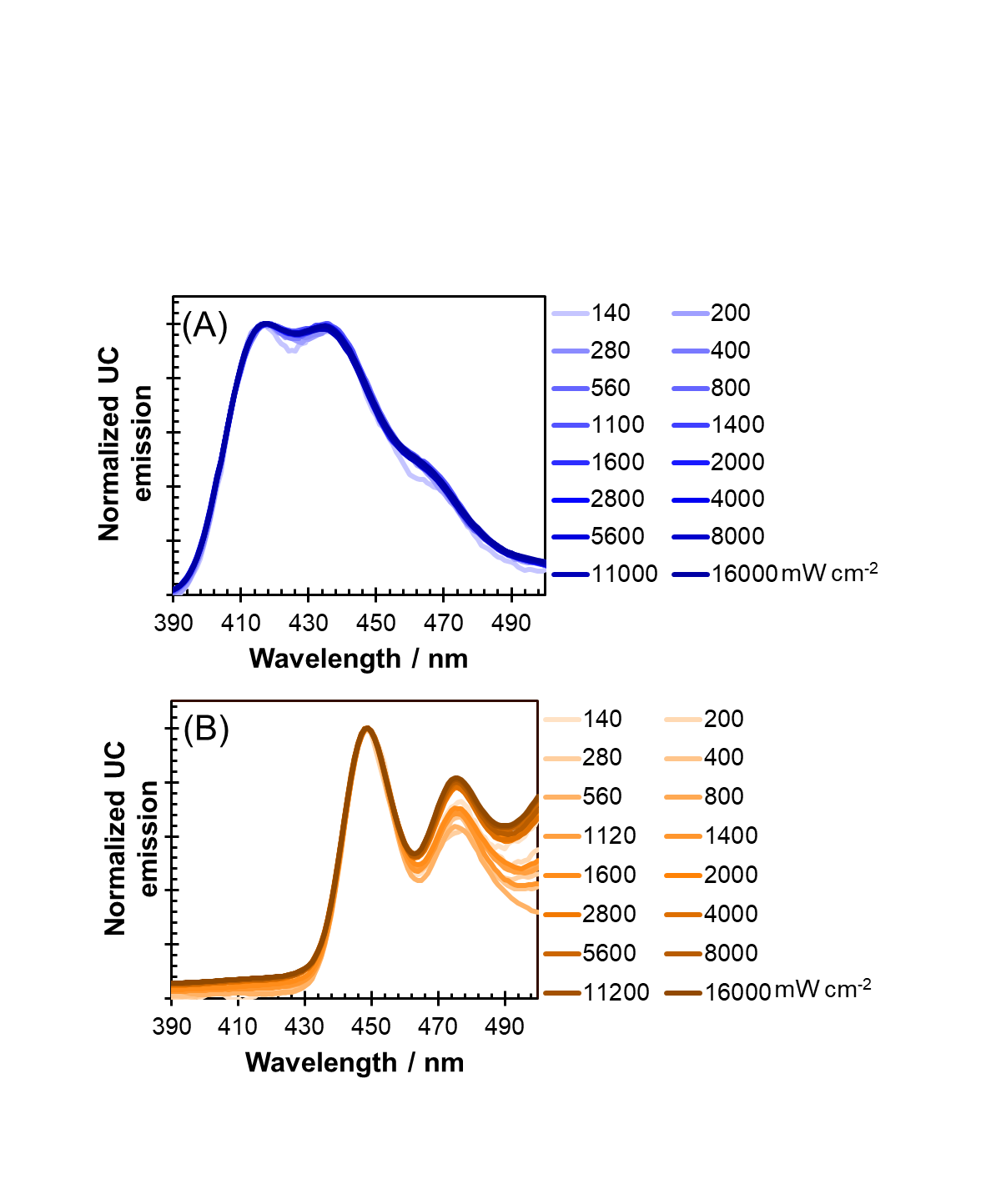


**Figure S3**. Excitation power (140-16000 mW cm^-2^) dependence of UC emission spectrum for (A) UC(DPA)/glass and (B) UC(TIPS)/glass.

**4. Absorption and emission spectra of solutions of TTA-UC elements**


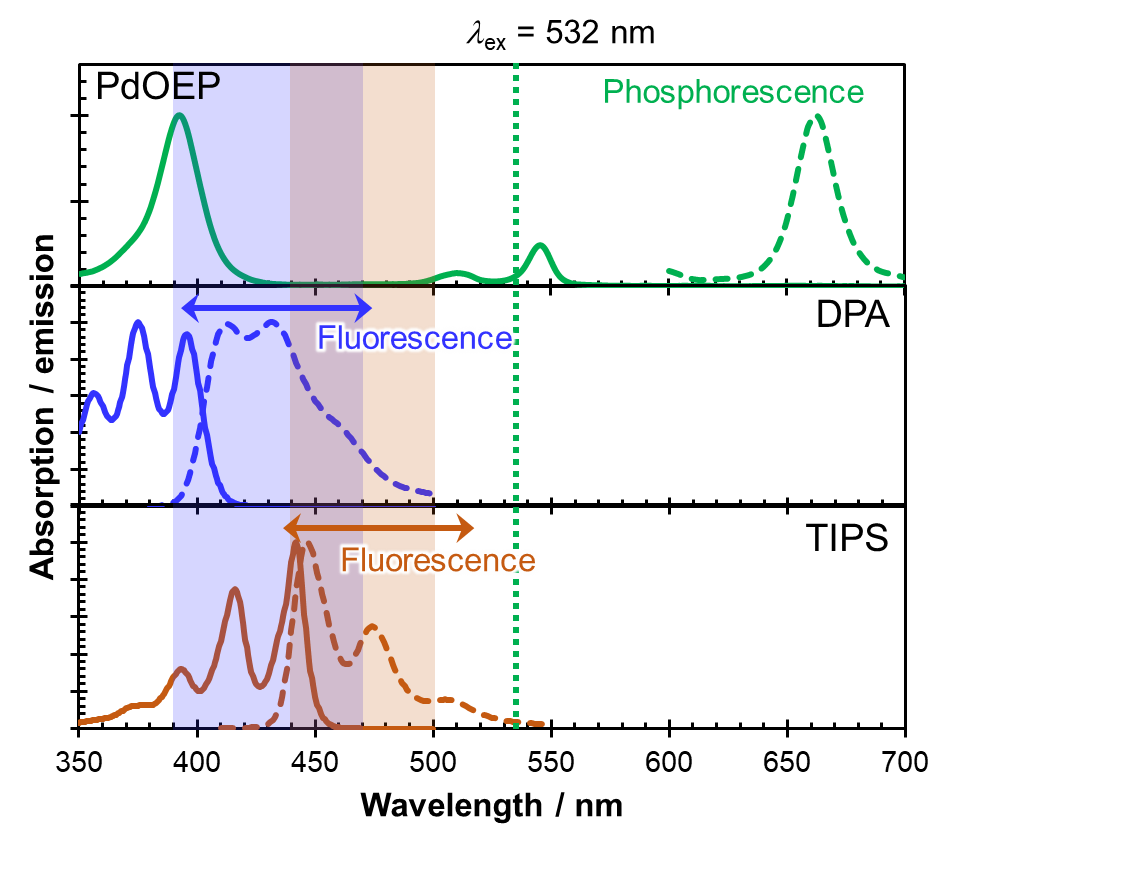


**Figure S4**. Absorption spectra (solid lines) and emission spectra (dashed lines) of solutions of PdOEP, DPA, and TIPS.

**5. The anti-Stokes emission efficiencies of UC(DPA)/glass and UC(TIPS)/glass.**

The anti-Stokes emission efficiencies ($\Phi_{\mathrm{UC}}$) for UC(DPA)/glass and UC(TIPS)/glass were determined using the following equation:

$$\Phi_{\mathrm{UC}}=\frac{1}{2}\Phi_{\mathrm{ISC}}\Phi_{\mathrm{TTET}}\Phi_{\mathrm{TTA}}f\Phi_{\mathrm{EM}} \left( S1 \right),$$

where $\Phi_{\mathrm{ISC}}$, $\Phi_{\mathrm{TTET}}$, $\Phi_{\mathrm{TTA}}$, and $\Phi_{\mathrm{EM}}$ represent the efficiencies of ISC of PdOEP, TTET from PdOEP to annihilators, TTA between the triplet-excited annihilators, and singlet emission of annihilators. Respective values for the parameters are summarized in **Table S1**.

**Table S1**. The values of parameters adopted to determine $\Phi_{\mathrm{UC}}$.

|  | $\Phi_{\mathrm{ISC}}$ | $\Phi_{\mathrm{TTET}}$ | $\Phi_{\mathrm{TTA}}$ | $f$ | $\Phi_{\mathrm{EM}}$ | $\Phi_{\mathrm{UC}}$ |
| --- | --- | --- | --- | --- | --- | --- |
| UC(DPA)/glass | 1 | 0.996 | 1 | 0.50 | 0.013 | 0.003 |
| UC(TIPS)/glass | 1 | 0.418 | 1 | 0.75 | 0.029 | 0.005 |

The factor $f$ refers to the generation probability of the singlet excited state after the TTA event and was cited from previous literature.^S1,S2^ The efficiency $\Phi_{\mathrm{ISC}}$ of PdOEP is generally known to be nearly 1.^S3^ To obtain the maximum UC yield, the saturated value of 1 was adopted for $\Phi_{\mathrm{TTA}}$, which depends on the excitation power. The $\Phi_{\mathrm{TTET}}$ values were obtained in this study ($\Phi_{\mathrm{TTET}}^{P-D}$: 0.996，$\Phi_{\mathrm{TTET}}^{P-T}$: 0.418). The $\Phi_{\mathrm{EM}}$ values for DPA and TIPS were experimentally determined as 0.013 and 0.029, respectively. From these values, $\Phi_{\mathrm{UC}}$ for UC(DPA)/glass and UC(TIPS)/glass were estimated to be 0.003 and 0.005, respectively.

The triplet lifetimes of DPA and TIPS in the mono-annihilator systems were obtained by fitting the time-resolved anti-Stokes emission measurements to equation S2.^S4^

$$I\left( t \right)\propto\left[ {}^{3}{E^{*}}\left( t \right) \right]^{2}=\left( \left[ {}^{3}{E^{*}} \right]_{0}\frac{1-\beta}{\exp\left( \frac{t}{\tau_{T}^{0}} \right)-\beta} \right)^{2} (S2),$$

where $I\left( t \right)$ is the anti-Stokes emission intensity at time *t*, $\left[ {}^{3}{E^{*}}\left( t \right) \right]$ and $\left[ {}^{3}{E^{*}} \right]_{0}$ are the triplet excited annihilator densities at time *t* and 0, respectively. The dimensionless parameter $\beta$ represents the decay efficiency by the TTA event from the annihilator triplet excited state and $\tau_{T}^{0}$ is the triplet lifetime of annihilator without the TTA event. The triplet exciton lifetimes of UC(DPA)/glass ($\tau_{T}^{0,D}$) and UC(TIPS)/glass ($\tau_{T}^{0,T}$), obtained from the time-resolved anti-Stokes emission measurements shown in **Figure S5**, were 4.1 ms and 1.1 ms, respectively. Additionally, the singlet exciton lifetimes for the annihilators in UC(DPA)/glass ($\tau_{S}^{0,D}$) and UC(TIPS)/glass ($\tau_{S}^{0,T}$) were estimated as 4.3 and 1.8 ns, respectively, from the time-resolved fluorescence measurements.


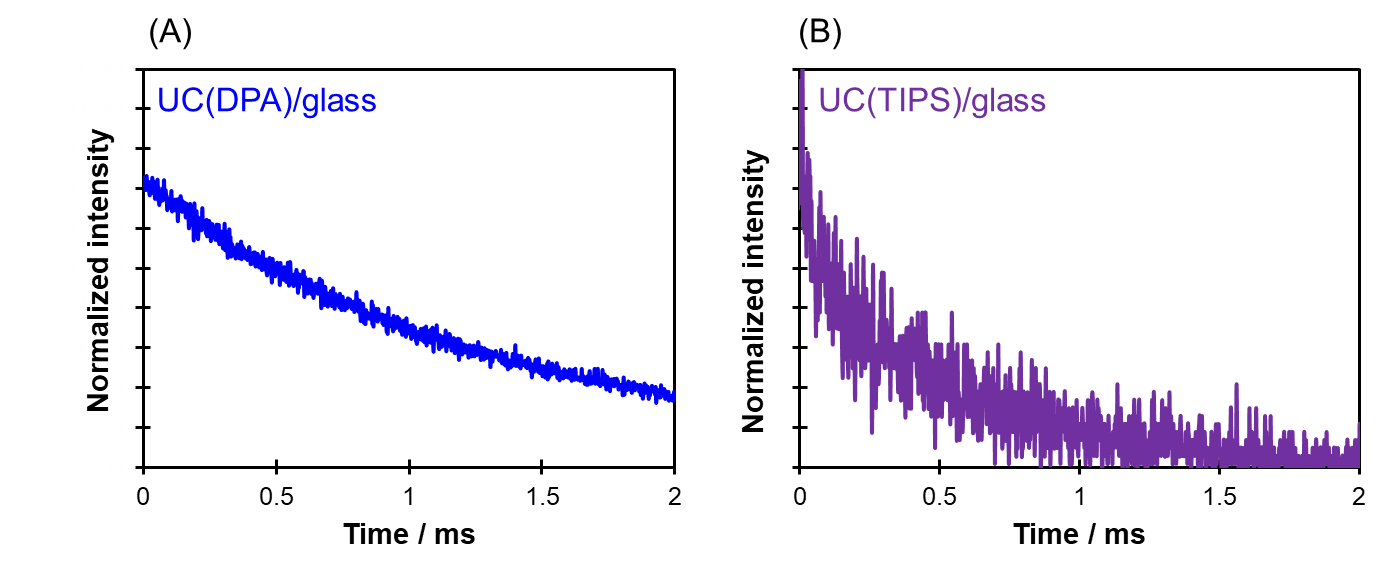


**Figure S5**. Time-resolved DPA-based anti-Stokes emission (*λ*_ex_ = 532 nm, 100 mW cm^-2^) in UC(DPA)/glass and TIPS-based anti-Stokes emission (*λ*_ex_ = 532 nm, 100 mW cm^-2^) in UC(TIPS)/glass. Anti-Stokes emissions of DPA and TIPS were detected at 420 and 480 nm, respectively.

**6. Phosphorescence spectra and calculation of TTET efficiencies of sample substrates**


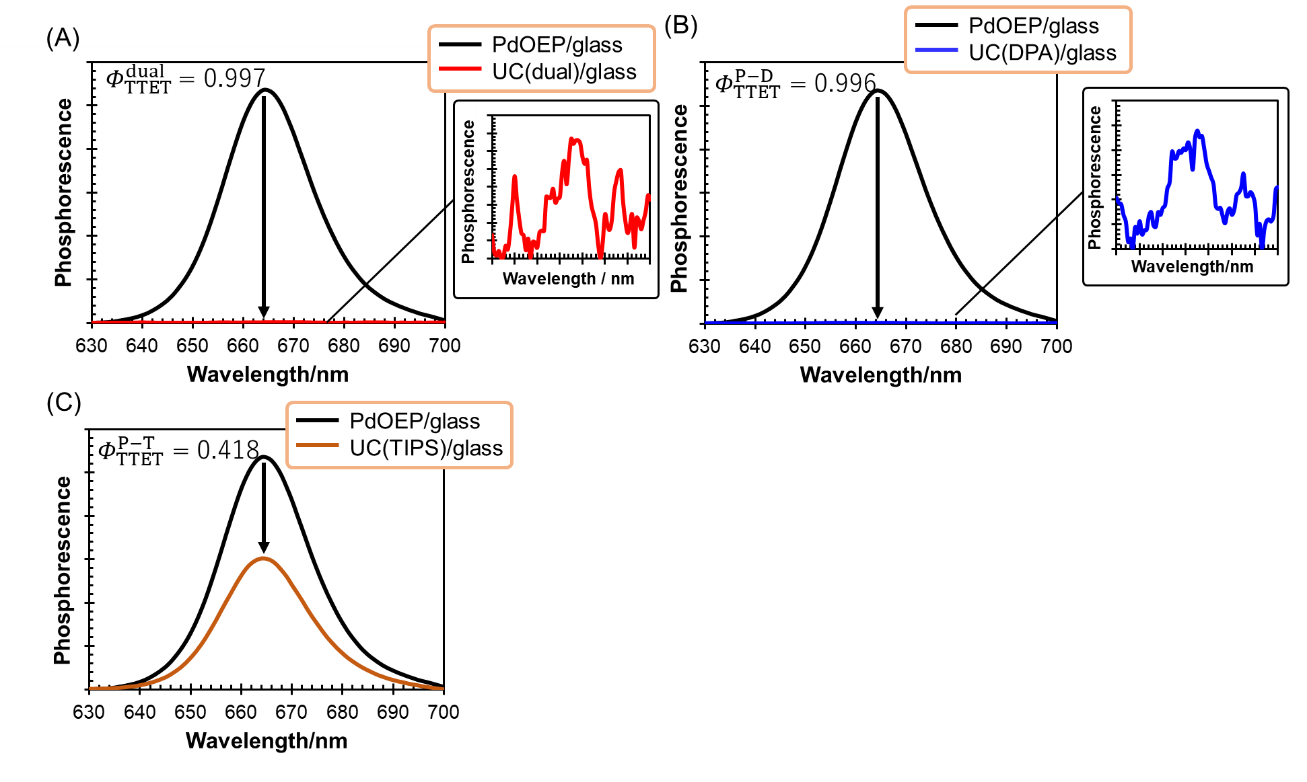


**Figure S6**. Phosphorescence spectra (*λ*_ex_ = 545 nm) of (A) UC(dual)/glass, (B) UC(DPA)/glass, and (C) UC(TIPS)/glass. The phosphorescence spectrum of PdOEP/glass is included in each figure for reference.

The TTET efficiency (*Φ*_TTET_) from triplet-excited sensitizer to annihilator for TTA-UC systems is generally described as follows.

$$\Phi_{\mathrm{TTET}}=\frac{k_{\mathrm{TTET}}}{k_{r}^{sens,T}+k_{\mathrm{nr}}^{sens,T}+k_{\mathrm{TTET}}}, \left( S1 \right)$$

where the $k_{r}^{sens,T}$, $k_{\mathrm{nr}}^{sens,T}$, and $k_{\mathrm{TTET}}$ are the rates of radiative and nonradiative decays and TTET from the triplet-excited sensitizer. Also, the phosphorescence efficiencies ($\Phi_{\mathrm{phos}}^{\mathrm{UC}}$ and $\Phi_{\mathrm{phos}}^{\mathrm{sens}}$) for the systems consisting of TTA-UC and sensitizer only are described as follows, respectively.

$$\Phi_{\mathrm{phos}}^{\mathrm{UC}}=\frac{k_{r}^{sens,T}}{k_{r}^{sens,T}+k_{\mathrm{nr}}^{sens,T}+k_{\mathrm{TTET}}} \left( S2 \right)$$

$$\Phi_{\mathrm{phos}}^{\mathrm{sens}}=\frac{k_{r}^{sens,T}}{k_{r}^{sens,T}+k_{\mathrm{nr}}^{sens,T}} \left( S3 \right)$$

$\Phi_{\mathrm{TTET}}$ is determined by Equation (S4), which is derived from Equations (S1)-(S3).

$$\Phi_{\mathrm{TTET}}=1-\frac{\Phi_{\mathrm{phos}}^{\mathrm{UC}}}{\Phi_{\mathrm{phos}}^{\mathrm{sens}}}=1-\frac{I_{\mathrm{phos}}^{\mathrm{UC}}}{I_{\mathrm{phos}}^{\mathrm{sens}}}, \left( S4 \right)$$

where $I_{\mathrm{phos}}^{\mathrm{UC}}$ and $I_{\mathrm{phos}}^{\mathrm{sens}}$ represent the phosphorescence intensities for systems consisting of TTA-UC and sensitizer only, respectively.

**Figure S6**(B) and (C) exhibit the phosphorescence spectra of UC(DPA)/glass and UC(TIPS)/glass, respectively. The phosphorescence spectrum of PdOEP/glass is also shown as a reference. In the present systems, the TTET efficiencies of UC(DPA)/glass ($\Phi_{\mathrm{TTET}}^{P-D}$) and UC(TIPS)/glass ($\Phi_{\mathrm{TTET}}^{P-T}$) are calculated as 0.996 and 0.418 using Equations (S5) and (S6) based on **Figure S6**(B) and (C), respectively.

$$\Phi_{\mathrm{TTET}}^{P-D}=\frac{k_{\mathrm{TTET}}^{P-D}}{k_{r}^{PdOEP,T}+k_{\mathrm{nr}}^{PdOEP,T}+k_{\mathrm{TTET}}^{P-D}}=1-\frac{I_{\mathrm{phos}}^{\mathrm{UC}\left( \mathrm{DPA} \right)}}{I_{\mathrm{phos}}^{\mathrm{PdOEP}}}, \left( S5 \right)$$

$$\Phi_{\mathrm{TTET}}^{P-T}=\frac{k_{\mathrm{TTET}}^{P-T}}{k_{r}^{PdOEP,T}+k_{\mathrm{nr}}^{PdOEP,T}+k_{\mathrm{TTET}}^{P-T}}=1-\frac{I_{\mathrm{phos}}^{\mathrm{UC}\left( \mathrm{TIPS} \right)}}{I_{\mathrm{phos}}^{\mathrm{PdOEP}}}, \left( S6 \right)$$

where $k_{r}^{PdOEP,T}$ and $k_{\mathrm{nr}}^{PdOEP,T}$ are the rates of radiative and nonradiative decays of PdOEP, respectively. The $k_{\mathrm{TTET}}^{P-D}$ and $k_{\mathrm{TTET}}^{P-T}$ are the TTET rates from PdOEP to DPA for UC(DPA)/glass and from PdOEP to TIPS for UC(TIPS)/glass, respectively. The $I_{\mathrm{phos}}^{\mathrm{PdOEP}}$, $I_{\mathrm{phos}}^{UC(DPA)}$, and $I_{\mathrm{phos}}^{UC(TIPS)}$ are the phosphorescence intensities of PdOEP/glass, UC(DPA)/glass, and UC(TIPS)/glass, respectively. Equations (S5) and (S6) correspond to Equation (1) in the main manuscript.

Assuming that TTET from PdOEP to DPA and TIPS in UC(dual)/glass occurred similarly to UC(DPA)/glass and UC(TIPS)/glass, respectively, the TTET efficiency ($\Phi_{\mathrm{TTET}}^{\mathrm{dual}}$) in UC(dual)/glass can be described as follows.

$$\Phi_{\mathrm{TTET}}^{\mathrm{dual}}=\frac{k_{\mathrm{TTET}}^{P-D}+k_{\mathrm{TTET}}^{P-T}}{k_{r}^{PdOEP,T}+k_{\mathrm{nr}}^{PdOEP,T}+k_{\mathrm{TTET}}^{P-D}+k_{\mathrm{TTET}}^{P-T}}=1-\frac{I_{\mathrm{phos}}^{\mathrm{UC}\left( \mathrm{dual} \right)}}{I_{\mathrm{phos}}^{\mathrm{PdOEP}}}, \left( S7 \right)$$

where $I_{\mathrm{phos}}^{UC(dual)}$ is the phosphorescence intensity of UC(dual)/glass. **Figure S6**(A) exhibits the phosphorescence spectra of UC(dual)/glass and PdOEP/glass as a reference. The $\Phi_{\mathrm{TTET}}^{\mathrm{dual}}$ is calculated as 0.997 using Equation (S7) based on **Figure S6**(A). In addition, the TTET efficiencies from PdOEP to DPA ($\Phi_{\mathrm{TTET}}^{P-D(dual)}$) and TIPS ($\Phi_{\mathrm{TTET}}^{P-T(dual)}$) in UC(dual)/glass are described by the following Equations, respectively.

$$\Phi_{\mathrm{TTET}}^{P-D(dual)}=\frac{k_{\mathrm{TTET}}^{P-D}}{k_{r}^{PdOEP,T}+k_{\mathrm{nr}}^{PdOEP,T}+k_{\mathrm{TTET}}^{P-D}+k_{\mathrm{TTET}}^{P-T}} \left( S8 \right)$$

$$\Phi_{\mathrm{TTET}}^{P-T(dual)}=\frac{k_{\mathrm{TTET}}^{P-T}}{k_{r}^{PdOEP,T}+k_{\mathrm{nr}}^{PdOEP,T}+k_{\mathrm{TTET}}^{P-D}+k_{\mathrm{TTET}}^{P-T}} \left( S9 \right)$$

The $\Phi_{\mathrm{TTET}}^{P-D(dual)}$ and $\Phi_{\mathrm{TTET}}^{P-T(dual)}$ are calculated as 0.968 and 0.029, respectively, using Equations (S5)-(S9) based on $\Phi_{\mathrm{TTET}}^{P-D}=0.996$, $\Phi_{\mathrm{TTET}}^{P-T}=0.418$, and $\Phi_{\mathrm{TTET}}^{\mathrm{dual}}=0.997$.

**7. Determination of concentration for TTA-UC components contained in UC(dual)/glass**

**
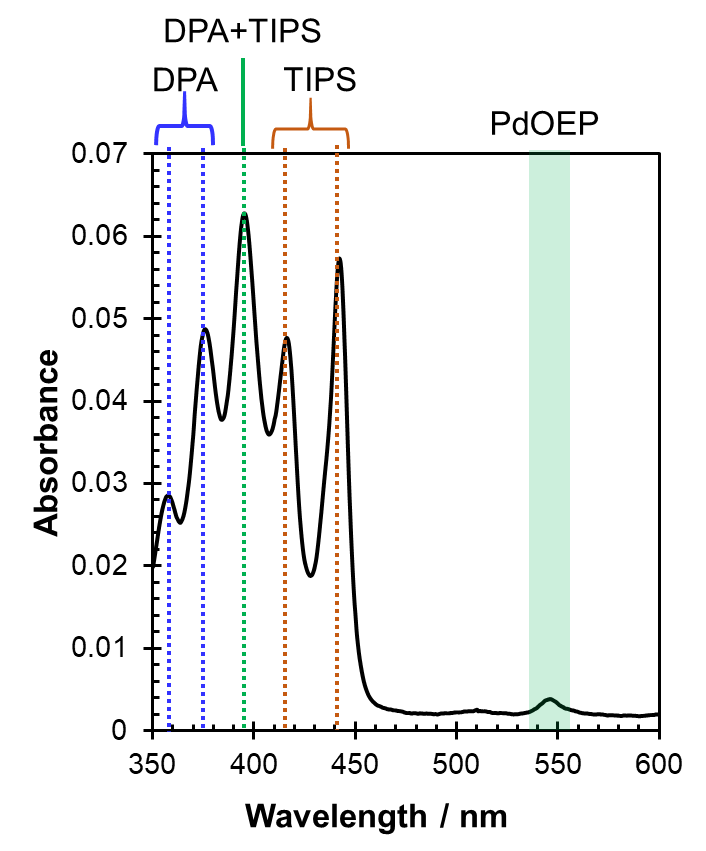
**

**Figure S7**. Absorption spectra of TTA-UC components dissolved in 1,2-dichloroethane.

To determine the concentrations of PdOEP, DPA, and TIPS contained in the TTA-UC thin films, each of the six UC(dual)/glass samples was sequentially immersed in 1,2-dichloroethane (3 mL) for 5 minutes to dissolve the TTA-UC components in the solvent. After confirming the dissolution of majority of TTA-UC components, the absorption spectrum of the solution containing these components was measured (**Figure S7**). Utilizing the absorbance values for PdOEP at 545 nm, DPA at 357 nm, and TIPS at 442 nm, concentrations of PdOEP, DPA, and TIPS in the TTA-UC thin film were estimated following the Lambert-Beer law as 11.5 mM, 380 mM, and 140 mM, respectively.

**8. Estimation of FRET^D-T^ efficiencies of UC(dual)/glass**


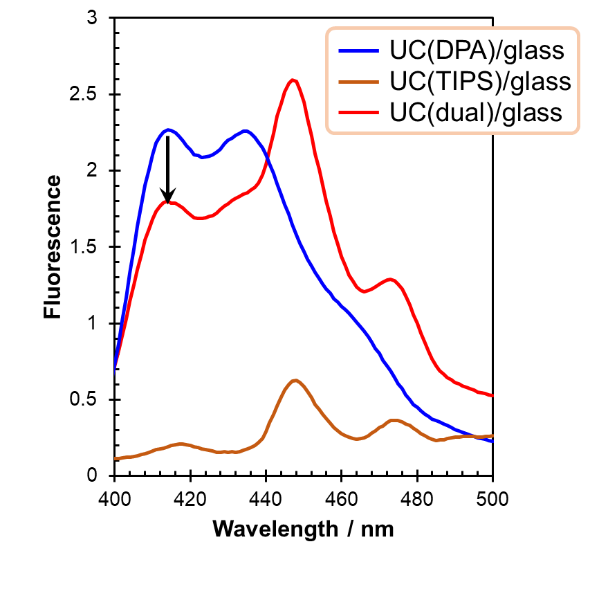


**Figure S8**. Fluorescence spectra (*λ*_ex_ = 358 nm) for UC(DPA)/glass, UC(TIPS)/glass, and UC(dual)/glass.

The annihilator(s) of UC(DPA)/glass, UC(TIPS)/glass, and UC(dual)/glass were directly excited at *λ*_ex_ = 358 nm (**Figure S8**). The resulting emission is attributed to the standard fluorescence process. UC(DPA)/glass and UC(TIPS)/glass exhibited only DPA and TIPS fluorescence, respectively. On UC(dual)/glass, both DPA and TIPS fluorescence were observed. It is worth noting that the DPA fluorescence intensity at 418 nm, where the TIPS fluorescence was negligibly small, was slightly lower than that of UC(DPA)/glass. The reduction suggests a marginal transfer of singlet-excited energy from DPA to TIPS through Förster-type singlet-singlet energy transfer. The efficiency ($\Phi_{\mathrm{FRET}}$) was estimated using the following equation:

$\Phi_{\mathrm{FRET}}=\left( 1-\frac{I_{\mathrm{fluo}}^{\mathrm{dual}}}{I_{\mathrm{fluo}}^{\mathrm{DPA}}} \right)\times100 \left( S10 \right),$

where $I_{\mathrm{fluo}}^{\mathrm{DPA}}$ and $I_{\mathrm{fluo}}^{\mathrm{dual}}$ represent the fluorescence intensities of DPA in UC(DPA)/glass and UC(dual)/glass, respectively, at 418 nm. The resulting $\Phi_{\mathrm{FRET}}$ was determined to be low, specifically 20.0 %.

**9. Estimation of the phosphorescence lifetime of PdOEP in UC(DPA)/glass**

From Equation S5, $\Phi_{\mathrm{TTET}}^{P-D}$ can be described as follows.

$$\Phi_{\mathrm{TTET}}^{P-D}=1-\frac{I_{\mathrm{phos}}^{\mathrm{UC}\left( \mathrm{DPA} \right)}}{I_{\mathrm{phos}}^{\mathrm{PdOEP}}}=1-\frac{\tau_{\mathrm{phos}}^{\mathrm{UC}\left( \mathrm{DPA} \right)}}{\tau_{\mathrm{phos}}^{\mathrm{PdOEP}}} \left( S11 \right),$$

where $\tau_{\mathrm{phos}}^{\mathrm{PdOEP}}$ and $\tau_{\mathrm{phos}}^{UC(DPA)}$ refer to the lifetimes of the triplet excited PdOEP in the absence and presence of the annihilator, respectively. The value of $\tau_{\mathrm{phos}}^{\mathrm{PdOEP}}$ (490 μs) was obtained from the time-resolved phosphorescence measurement on PdOEP/glass. The phosphorescence lifetime $\tau_{\mathrm{phos}}^{UC(DPA)}$ in UC(DPA)/glass was estimated to be 2 μs from the following equation:

$$\tau_{\mathrm{phos}}^{\mathrm{UC}\left( \mathrm{DPA} \right)}=\left( 1-\Phi_{\mathrm{TTET}}^{P-D} \right)\tau_{\mathrm{phos}}^{\mathrm{PdOEP}}=\left( 1-0.996 \right)\times490 \mu s\cong2 \mu s \left( S12 \right).$$

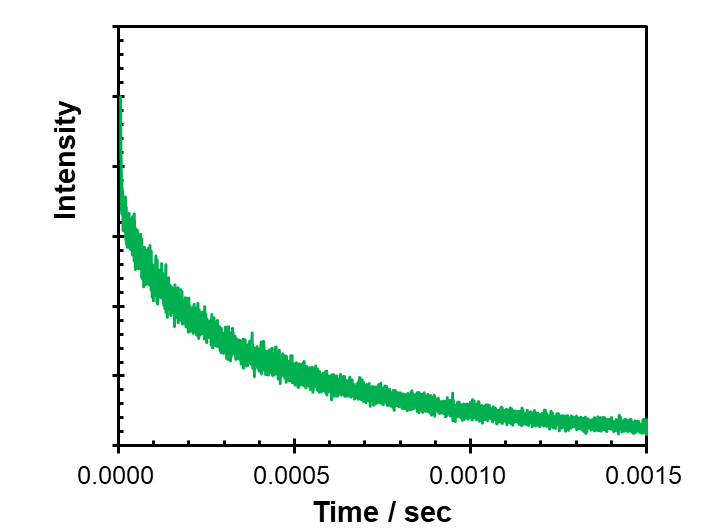


**Figure S9**. Time-resolved phosphorescence measurement (*λ*_ex_ = 532 nm) of PdOEP of PdOEP/glass.

**10. Time-resolved TIPS-based anti-Stokes emission in UC(dual)/glass**


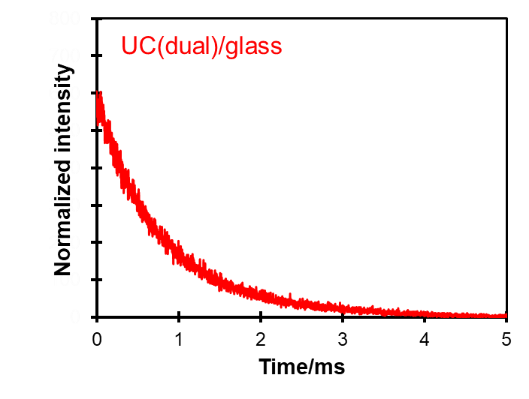


**Figure S10**. Time-resolved TIPS-based anti-Stokes emission (*λ*_ex_ = 532 nm) in UC(dual)/glass with an excitation power of 100 mW cm^-2^. The emission was detected at 480 nm.

**11. Phosphorescence spectra of UC(dual)/AgPRs and UC(dual)/glass**


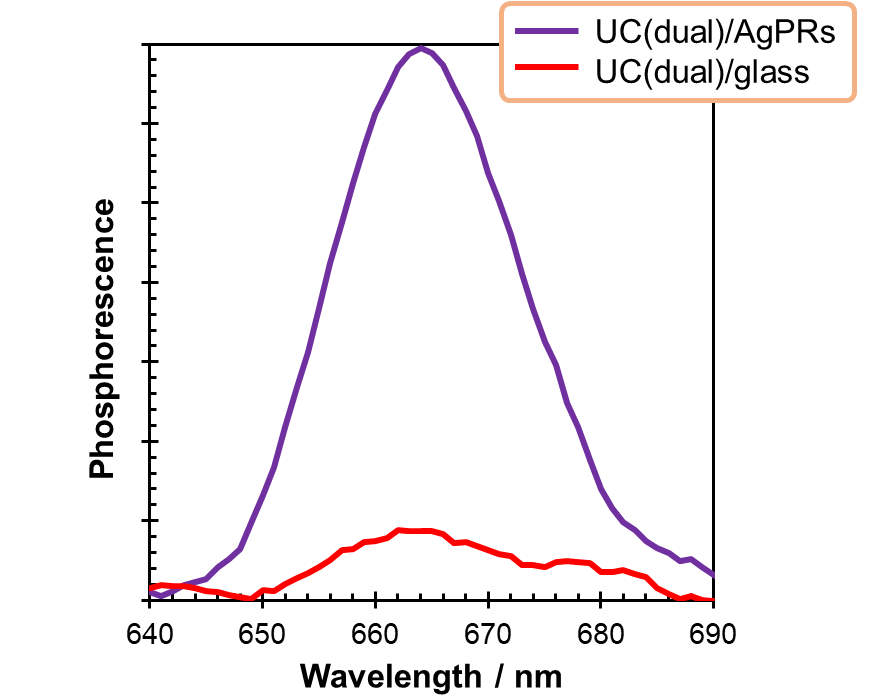


**Figure S11**. Phosphorescence spectra of (*λ*_ex_ = 545 nm) for UC(dual)/AgPRs and UC(dual)/glass.

**12. Geometric models of AgPRs used in BEM calculations**


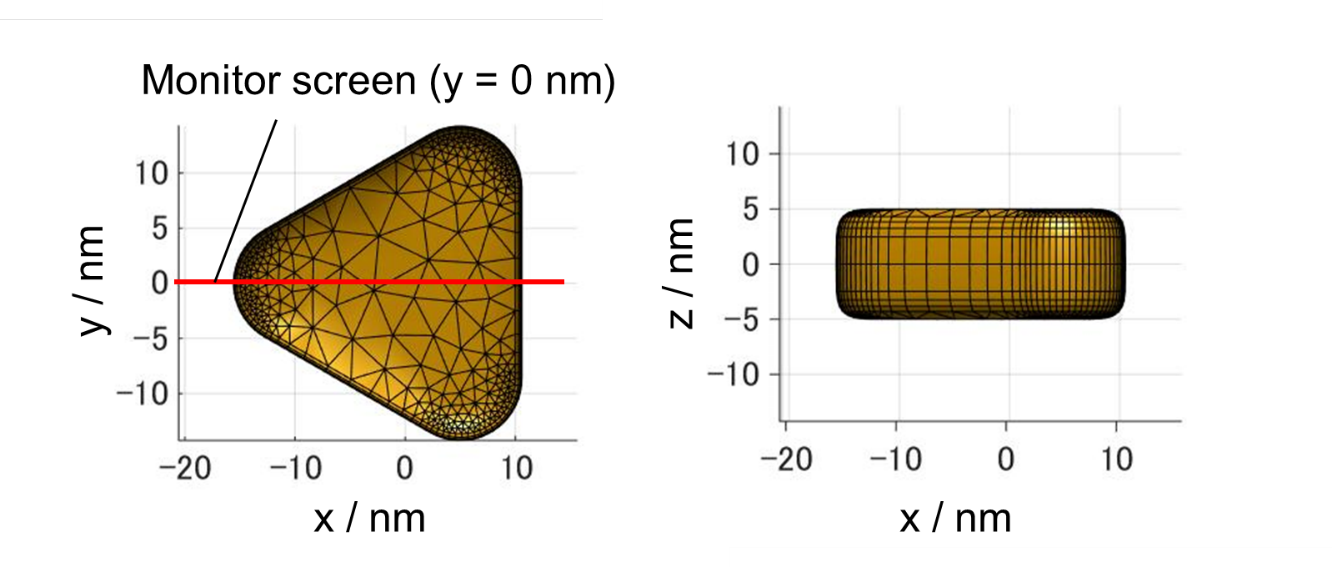


**Figure S12**. Geometric models of AgPRs used in the calculation of local electromagnetic field distribution using BEM (surrounding medium: refractive index: 1.48).^S5^

**References**

S1. C. Gao, S. K. K. Prasad, B. Zhang, M. Dvořák, M. J. Y. Tayebjee, D. R. McCamey, T. W. Schmidt, T. A. Smith, W. W. H. Wong, Intramolecular Versus Intermolecular Triplet Fusion in Multichromophoric Photochemical Upconversion, *J. Phys. Chem. C.,* 2019, **123**, 20181-20187.

S2. N. Nishimura, V. Gray, J. R. Allardice, Z. Zhang, A. Pershin, D. Beljonne, A. Rao, Photon Upconversion from Near-Infrared to Blue Light with TIPS-Anthracene as an Eﬃcient Triplet−Triplet Annihilator *ACS Materials Lett*., 2019, **1**, 660-664.

S3 K. N. Solov’ev, P. M. Tsvirko, V. V. Spunov, Quantum yield for intersystem crossing in nonfluorescent metal porphyrins. *J. Appl. Spectrosc*. *1973*, **18**, 543-545.

S4 F. Edhborg, A. Olesund, B. Albinsson, Best practice in determining key photophysical parameters in triplet-triplet annihilation photon upconversion. *Photochem. Photobiol. Sci*. *2022*, **21**, 1143-1158.

S5. J. Waxenegger, A. Trügler, U. Hohenester, Plasmonics Simulations with the MNPBEM Toolbox:

Consideration of Substrates and Layer Structures, *Comput. Phys. Commun.*, 2015, **193**, 138–150.
